# Reducing genomic instability in iPSCs

**DOI:** 10.18632/oncotarget.6212

**Published:** 2015-10-21

**Authors:** Sergio Ruiz, Oscar Fernandez-Capetillo

**Affiliations:** Genomic Instability Group, Spanish National Cancer Research Centre, Madrid, Spain

**Keywords:** cell reprogramming, nucleosides, replication stress, genomic instability

Over the past 15 years, pluripotent cells, either embryonic stem cells (ESCs) or induced pluripotent stem cells (iPSCs), became a formidable cellular platform to study developmental biology, model disease *in vitro* and pursue new drug discoveries. Furthermore, the promise of using endless cell derivatives from pluripotent cells to treat incurable diseases such as diabetes, neurodegenerative or heart diseases, holds hope for the new wave of coming regenerative medicine procedures [1]. However, although different clinical trials are currently ongoing by using pluripotent cell derivatives, major concerns regarding the genomic instability of ESCs and especially iPSCs, which results in the accumulation of genomic aberrations, have hampered a broader clinical application.

Genomic instability in pluripotent cells has been detected since the early 2000's when karyotypically abnormal human ESC lines were observed following their derivation and expansion in culture. The induction of pluripotency by reprogramming somatic cells into iPSCs through the forced expression of defined transcription factors has also been associated with the accumulation of genomic aberrancies ranging from whole chromosome aneuploidies, sub-chromosomal deletions/amplifications or copy number variants (CNVs) to point mutations [2]. However, the reasons underlying the genomic instability observed in pluripotent cells remained elusive.

**Figure 1:**
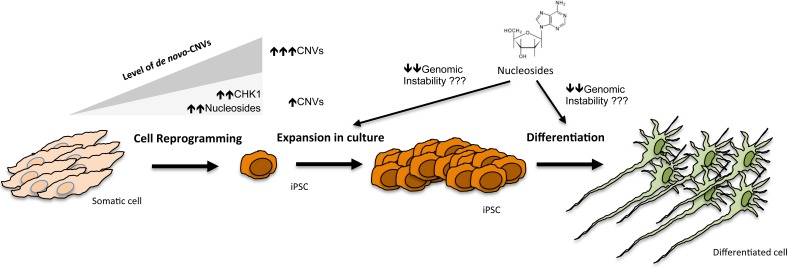
Induction of pluripotency in somatic cells by cell reprogramming induces genomic instability and leads to the accumulation of *de novo*-CNVs This figure illustrates the different steps at which genomic rearrangements might arise during the generation or use of iPSCs. However, the amount of these RS-induced newly generated CNVs can be lowered by elevating the expression of RS-chekpoint kinases, such as CHK1, or by supplementing the culture media with additional nucleosides. It would be interesting to evaluate whether addition of nucleosides could also limit the level of genomic instability in iPSCs after expansion in culture or differentiation towards specific differentiated cell types.

Replication stress (RS) is a source of DNA damage associated with the stalling of replication forks, which leads to the accumulation of ssDNA that in turn causes recombination and genomic rearrangements. In the context of cancer, previous studies had shown that oncogene-induced RS is a major cause of the genomic instability observed in tumor cells [3]. We have now reported that, similar to oncogenes, reprogramming factors also generate RS, which contributes significantly to the genomic instability observed in iPSCs [4]. Supporting this view, up to 70% of the CNVs that were generated *de novo* during reprogramming mapped to regions previously found to undergo rearrangements in response to RS [4]. The causes behind reprogramming-induced RS are still not fully understood, but might have to do with the sudden change in the replication rate that occurs from a differentiated cell (slower replication depending on few origins) to a pluripotent stem cell (fast replication triggered by many origins). This might create imbalances such as insufficient amount of nucleotides, or limiting amounts of RS-checkpoint proteins that cannot cope with the enhanced exposure of ssDNA. Accordingly, overexpression of the CHK1 kinase (a major responder to RS) or supplementation of the reprogramming culture media with nucleosides significantly decreased genomic instability in the resultant iPSCs (Figure 1) [4].

Noteworthy, genomic instability in iPSCs might be influenced by factors beyond reprogramming [5]. In fact, besides the genomic rearrangements that can arise due to the reprogramming process, pre-existing abnormalities in the somatic cell of origin might become fixed in the population after clonal isolation of iPSCs. In addition, new rearrangements can arise during the long-term *in vitro* expansion of iPSC (or ESC) cultures, due to the intrinsic propensity of rapidly proliferating stem cells to undergo genomic rearrangements. Regardless of the origin of the genomic aberrations that are observed in pluripotent cells, there remains the reasonable doubt that some of these can have undesirable consequences affecting the functionality of the cells, their ability to self-renew or to differentiate into specific cell types. Moreover, given that many of the genomic aberrations land on fragile sites that are also frequently found rearranged in cancer, this raises an additional flag on the potential tumorigenic impact of some of these mutations. Hence, whereas the impact of these mutations remains to be formally proven, implementing procedures to obtain iPSCs with lower genomic instability would be desirable.

Due to the high proliferation rates required for successful reprogramming and the fast cell cycle kinetics existing in pluripotent cells in culture [6], the accumulation of RS is inherently associated with the pluripotent state. Nonetheless, as we have shown in our work, a simple procedure such as the supplementation of the culture media with a supply of nucleosides serves to significantly diminish reprogramming-induced RS and genomic instability in iPSCs [4]. It remains to be seen whether addition of nucleosides can also limit the genomic instability associated with the *in vitro* expansion of pluripotent cells or after differentiation into different cell types (Figure 1).

The issue of genetic instability in iPSC lines is a very timely and relevant issue that needs to be addressed. In fact, the control of genomic instability in pluripotent stem cells is probably one of the major hurdles in the race to implement stem cell technologies in the clinic [7]. Understanding how genomic aberrancies accumulate in ESCs and iPSCs could help towards the goal of translating this technology into patients. Our work showed that only the addition of nucleosides to the culture media during reprogramming mitigated the impact of genomic instability and seems a simple practice to implement not only for the generation of new iPSC lines, but also for the general culture techniques of pluripotent stem cells.
